# Hepatitis B prevention and treatment needs in women in Senegal (ANRS 12356 AmBASS survey)

**DOI:** 10.1186/s12889-023-15710-y

**Published:** 2023-05-05

**Authors:** Tchadine Djaogol, Lauren Périères, Fabienne Marcellin, Assane Diouf, Maria Patrizia Carrieri, Aldiouma Diallo, Sylvie Boyer, for the ANRS 12356 AmBASS Study Group, Cyril Bérenger, Marwan al Qays Bousmah, Morgane Bureau, Patrizia Carrieri, Marion Coste, Maëlle de Seze, Gwenaëlle Maradan, Carole Treibich, Elhadji Ba, Fambaye Dièye, Elhadji Bilal Faye, Assane Ndiaye, Cheikh Sokhna, Mouhamadou Baba Sow, Coumba Touré Kane, Gora Lo, Anna Julienne Selbé Ndiaye, Samba Ndiour, Philippe Halfon, Sofiane Mohamed, Nicolas Rouveau, Maria-Camila Calvo Cortès, Gabrièle Laborde-Balen, Martine Audibert, Fatou Fall, Ibrahima Gueye, Karine Lacombe, Moussa Seydi, Yusuke Shimakawa, Edouard Tuaillon, Muriel Vray

**Affiliations:** 1 Aix Marseille Univ, Inserm, IRD, SESSTIM, Sciences Economiques & Sociales de La Santé & Traitement de L’Information Médicale, ISSPAM, Marseille, France; 2 Univ. Bordeaux, INSERM, Institut Bergonié, BPH, U1219, CIC-P 1401, F-33000 Bordeaux, France; 3 VITROME, Campus IRD-UCAD, Dakar, Senegal

**Keywords:** Hepatitis B, Prevention, Treatment, Mother to child transmission, Senegal, West Africa

## Abstract

**Background:**

Although mother-to-child transmission (MTCT) of hepatitis B virus (HBV) is prevalent in West Africa, epidemiological data on HBV infection in women remain scarce. We studied i) hepatitis B surface antigen (HBsAg) prevalence and its correlates, ii) HBV screening history and serological status awareness, iii) MTCT risk and treatment needs in Senegalese women.

**Methods:**

A cross-sectional population-based serosurvey for HBsAg positivity was conducted in 2018–2019 in the rural area of Niakhar (Fatick region, Senegal). Participants were offered home-based HBV screening and answered face-to-face questionnaires. HBsAg-positive participants underwent clinical and biological assessments. Data were weighted and calibrated to be representative of the area’s population. Logistic regression models helped identify factors associated with HBsAg-positivity in adult women (> 15 years old).

**Results:**

HBsAg prevalence in adult women was 9.2% [95% confidence interval: 7.0–11.4]. Factors associated with HBsAg-positivity were being 15–49 years old (ref: ≥ 50), living in a household with > 2 other HBsAg-positive members, and knowing someone with liver disease. Only 1.6% of women had already been tested for HBV; no one who tested HBsAg positive was already aware of their serological status. In women 15–49 years old, 5% risked MTCT and none were eligible for long-term antiviral treatment.

**Conclusions:**

Adult women have a high HBsAg prevalence but a low MTCT risk. Low rates of HBV screening and serological status awareness argue for the adoption of systematic screening during pregnancy using free and rapid diagnostic tests. Additionally, screening household members of HBsAg-positive women may greatly improve the cascade of care in rural Senegal.

**Trial registration:**

ClinicalTrials.gov identifier (NCT number): NCT03215732.

**Supplementary Information:**

The online version contains supplementary material available at 10.1186/s12889-023-15710-y.

## Background

Without timely diagnosis and adequate treatment, chronic hepatitis B virus (HBV) infection can progress to liver fibrosis, cirrhosis, and hepatocellular carcinoma (HCC) [1]. In Sub-Saharan Africa (SSA), HBV prevalence is above 8% and related morbidity and mortality are particularly high due to limited access to testing and antiviral treatment [2–4]. West Africa is the most affected area with 31,000 HCC-related deaths in 2015 [5, 6].

In SSA, exposure to HBV predominantly occurs during early childhood, mostly through horizontal and mother-to-child transmission (MTCT), and HBV infection is one of the most common perinatally acquired infectious diseases [7]. HBV infection through MTCT is associated with a 90% risk of developing chronic infection [8, 9] and with a fivefold higher risk of developing liver complications, including cirrhosis and HCC, than horizontal transmission [6]. Prior to the introduction of HBV immunization, MTCT accounted for 10% of all HBV infections in this region [10]. However, the relative contribution of MTCT to new HBV infections is expected to increase in the near future due to increased pentavalent vaccine coverage (including HBV) in children, without the implementation of additional MTCT preventive measures [11].

Preventing HBV MTCT in SSA is therefore indispensable if the World Health Organization’s (WHO) target of hepatitis B elimination by 2030 is to be achieved [7]. Three key interventions are recommended in the WHO’s 2020 guidelines for the prevention of MTCT: [8] i) a first dose of HBV vaccine within 24 h after birth to all newborns (‘birth dose’ hereafter); ii) HBV screening for all pregnant women; iii) short-term treatment with Tenofovir (from the 28^th^ week of pregnancy until at least delivery) for pregnant women at high risk of MTCT, defined as an HBV DNA ≥ 200,000 IU/mL or hepatitis B e antigen (HBeAg) positivity (in settings where HBV DNA quantification is unavailable) [8].

In Senegal, HBV infection is highly endemic with an estimated prevalence of 6.9% (95% confidence interval (CI) 5.6–8.1%) in rural areas [12]. To prevent HBV MTCT, the birth dose was introduced into the Expanded Program on Immunization in 2016 to complement the existing (since 2004) pentavalent combine vaccine scheduled at 6, 10 and 14 weeks after birth. HBV screening is recommended in pregnant women but is not included in the free tests offered during antenatal consultations. The national hepatitis program plans to decentralize HBV screening, care and treatment services at the regional and district levels of the healthcare system [13]. However, access to treatment remains limited for the prevention of MTCT in pregnant women at high risk.

Preventing HBV MTCT is particularly challenging in rural areas, where access to healthcare services is limited. Epidemiological data on HBV infection and MTCT risk in women living in West Africa remain scare [14, 15]. However, such data are required to assess HBV prevention, care and treatment needs in this population, and to design and implement effective prevention interventions.

We aimed to study the following in adult rural Senegalese women: i) chronic HBV infection prevalence and its correlates; ii) their knowledge of HBV infection, HBV screening experience and awareness of HBV their serological status; iii) HBV MTCT risk and treatment needs.

## Methods

### Study setting

ANRS 12356 AmBASS is a large population-based cross-sectional survey conducted from October 2018 to May 2019 to assess the socioeconomic and public health impact of chronic HBV infection in the rural area of the Niakhar Health and Demographic Surveillance System (HDSS), in the region of Fatick in Senegal [16].

The Niakhar HDSS comprises 44,854 inhabitants living in 30 villages over a 203 km^2^ area [17]. The economy of this rural area is mainly based on agriculture and livestock farming [18]. Four primary healthcare posts managed by nurses act as first-contact facilities offering basic services in the area, including maternal and child healthcare as well as normal deliveries (i.e., no caesarian section, etc.). Furthermore, two healthcare centres managed by physicians (reference facilities at the district level) and the regional hospital are located close to the HDSS area [18].

The study area is of particular interest as the Fatick region was selected in 2018 by the National Hepatitis Program to be the pilot region for the decentralization of HBV care.

### Study design and participants

Two-stage stratified sampling was used to select participating households [16, 19]. The required sample size (*n* = 3,200 participants) was calculated to provide an estimation of the HBs antigen (HBsAg) prevalence in the general population and in women of childbearing age (WCBA) (15–49 years) with a precision of ± 1.2% and ± 3%, respectively. We hypothesized a minimum average prevalence of 10% and a maximum of 17% based on previous findings, as described more in detail in the study protocol [16].

All individuals aged ≥ 6 months who lived (or intended to live) for at least 6 months during the year in the selected households were invited to participate. Informed consent to participate was mandatory; parental consent was obtained for all participating minors. The full design of the survey is presented in detail elsewhere [16].

### Ethical considerations

The study received ethical approval from the Senegalese National Ethical Committee for Research in Health (no. 082MSAS/DPRS/CNERS), and authorization from the French Commission on Information Technology and Liberties (reference MMS/HG/OTB/AR181521). The study conforms to the declaration of Helsinki.

### Data collection

#### Data collection at the home level

Household members who agreed to participate received pre-HBV test counseling and were then screened by nurses using dried blood spots (DBS, Whatman 903 Protein saver card). This simple and reliable technique (diagnostic sensitivity and specificity > 90%) has been recommended by the WHO to detect HBsAg in areas where rapid diagnostic tests (RDT) are not available or where there are no facilities or expertise to take venous blood specimens [20].

After screening, face-to-face questionnaires were conducted to document participants’ socio-demographic and socioeconomic characteristics, maternal health (for WCBA), HBV screening history, and general knowledge of hepatitis B.

Another questionnaire was also administered to the head of the household or its representative, to document housing characteristics and household resources including durable goods, agricultural and farming resources.

#### Clinical and biological data collection at the healthcare facility level

All participants were informed of their HBV screening test results and received post-test counselling. HBsAg-positive participants were offered a consultation with the study physician for additional clinical and biological examinations to assess liver disease stage and treatment eligibility. Venous blood samples were collected for full blood count, alanine aminotransferase (ALT), aspartate aminotransferase (AST), HBeAg, antibody to HIV (ARCHITECT, Abbott), and antibody to hepatitis D virus (HDV) (Enzyme Linked Immunosorbent Assay, Hepatitis Delta, Virion\Sirion). HBV DNA levels were assessed (RT-PCR, Gene Proof DNA, Biosynex) using DBS.

#### Laboratory procedures

All DBS and blood samples were transported to the Niakhar station laboratory where they were stored and transferred weekly to the Institute for Health Research, Epidemiological Surveillance and Training laboratory for analysis. Using a standardized method [21], the DBS were eluted to detect HBsAg using a chemiluminescent microparticle immunoassay (ARCHITECT, Abbott, Sligo, Ireland). Based on the results of the AmBASS pilot study, which collected 39 paired DBS and serum samples, we defined HBsAg cut-off values at 1.0 IU/mL (for negativity) and 1.5 IU/mL (for positivity) using DBS (Additional file 1). A second HBsAg test was systematically performed using venous blood sampling for participants with HBsAg values between 1.0 and 1.5 IU/mL on DBS [16].

### Study population

For the present study, the study population comprised all adult women (> 15 years old) who participated in the AmBASS survey. We also analyzed the two following subgroups in this population: (i) WCBA, and (ii) WCBA with at least one full-term pregnancy.

### Statistical analyses

#### Data weighting and calibration

Participants’ data were weighted and calibrated to ensure that the study population was representative of all adult women of the Niakhar HDSS in terms of age (Additional file 2). All analyses were performed using weighted and calibrated data. Sampling weights were calculated as the inverse of the individual probability of inclusion in the sample, divided by the number of months each individual in the household was present during the preceding year. The following variables were used: number of participants in the household, number of eligible individuals in the household, number of households participating in the survey in the given village, number of randomly selected households in the given village, number of villages randomly selected for the survey, number of villages in the Niakhar HDSS.

#### Descriptive analyses

The characteristics of the whole study population and of the two subgroups were described using percentages for categorical variables and mean ± standard deviation (SD) for continuous variables. We compared the characteristics of WCBA (15–49 years) with those of older women (aged ≥ 50 years) using Wilcoxon Mann–Whitney test and Chi2 test for continuous and categorical variables, respectively.

#### Prevalence estimations

Chronic HBV infection was defined by HBsAg positivity based on a single assessment according to the WHO’s recommendation for settings where HBsAg seroprevalence is above 0.4% [20]. HBsAg positivity prevalence was calculated for all adult women and according to age class (15–49 years old; ≥ 50 years old). The prevalence 95% confidence intervals were estimated using standard Wald confidence limits for proportions.

#### Factors associated with chronic HBV infection

Logistic regression models were performed to identify factors associated with chronic HBV infection in the study population. Factors with a *p*-value < 0.25 in univariable analyses were considered eligible for multivariable analysis. The final multivariable model was built using a backward stepwise selection procedure, with a *p*-value threshold set at 0.10.

#### HBV MTCT risks and antiviral treatment needs

A high risk of HBV MTCT (requiring antiviral prophylaxis during pregnancy) was defined as having HBV DNA ≥ 200,000 IU/mL [8].

Long-term treatment eligibility was assessed using WHO guidelines, which recommend treatment initiation for the following conditions: (i) clinical evidence of cirrhosis or (ii) AST-to-platelet ratio index (APRI) score above two or (iii) age ≥ 30 years old with persistently abnormal ALT levels and HBV DNA > 20,000 IU/mL [22]. We applied the ALT value measured at a single time point and considered an upper limit of normal (ULN) value of 19 U/L [22].

All analyses were performed using STATA, version 14.2 for Windows (StataCorp, College Station, USA).

## Results

### Characteristics of the study population

#### Socio-demographic and socioeconomic characteristics

**Fig. 1:**
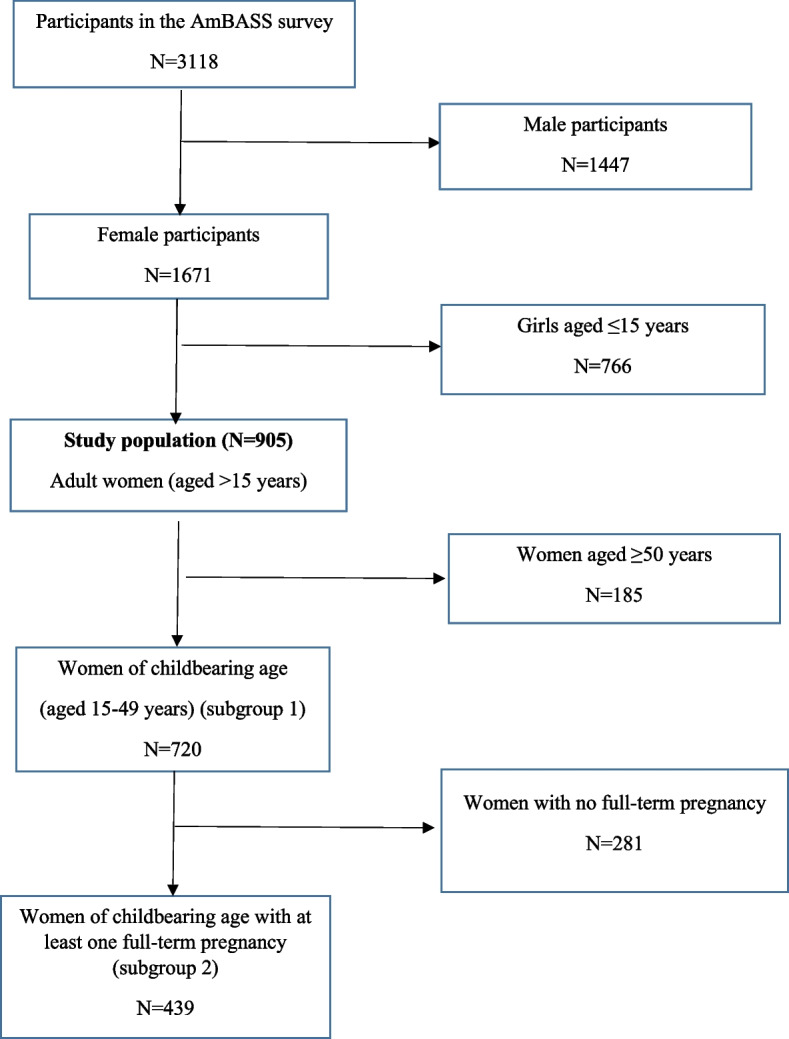
Selection of the study population and of the two subgroups of women considered in the analyses (i) and (ii)) (ANRS 12356 AmBASS survey)

Among the 3,118 AmBASS participants, 1671 (54.0%) were women, 905 (54.0%) of whom were aged > 15 years (study population). Of the latter, 720 (80.0%) were WCBA, including 439 (60.0%) women who already had one full-term pregnancy (Fig. 1).

Mean age (SD) of the women in the study population was 33.9 (16.3) years (Table 1). Two-thirds (61.8%) were married and 44.2% had at least four children. Approximately half (51.6%) had never attended school, 31.8% were studying and 58.9% had an economic activity, mainly in the farming sector.

**Table 1:** Characteristics of adult women (*n* = 905) (ANRS 12356 AmBASS, weighted and calibrated data)

**Variables** (% of missing data in adult women and in WCBA)	**Adult women (> 15 years)** ***n* = 905**
**Socio-demographic and socioeconomic characteristics**	
**Age** (in years) (0.0; 0.0)	34.3 (16.3)
**Matrimonial status** (1.5; 1.9**)**	
Married in a polygamous union	44.9
Married in a monogamous union	16.9
Not married	38.2
**Total number of children** (1.8; 1.9)	3.2 (3.2)
**Number of children** (1.8; 1.9)	
0	34.9
1–3	20.9
≥ 4	44.2
**Area of residence** (0.0; 0.0)	
Semi-urban	64.5
Rural	35.5
**Educational level** (2.2; 2.4)	
Junior high school or above	34.8
Primary or below	13.6
Never attended school	51.6
**Activity** (1.8; 2.2)	
Inactive	9.3
Farming and/or non-farming activity	58.9
Studies/training	31.8
**Household wealth index** (0.0; 0.0) ^a^	
1^st^ quartile	17.5
2^nd^ quartile	21.8
3^rd^ quartile	23.6
4^th^ quartile	37.1
**Having community health insurance** (1.5; 1.7)	
Yes	4.5
No	95.5
**HBV screening**	
**Living in a household with at least two other HBsAg-positive individuals (**0.0; 0.0)	5.8
**Having been previously tested for HBV** (2.4; 2.8)	
Yes	1.6
No	98.4
**Awareness of HBV serostatus among women previously tested** (0.0; 0.0)	***n* = 8**
Yes, positive	0.0
Yes, negative	95.5
Do not know	4.5
**Reasons for never having been tested for HBV**	***n* = 875**
Never heard of HBV testing (0.0; 0.0)	83.3
Not offered in the antenatal sessions (0.0; 0.0)	8.4
**Knowledge of hepatitis B**	
**Are you aware of the liver diseases, which we call ‘big belly’ and ‘yellow eyes’?** (1.4; 1.7)	
Yes	22.3
No	77.7
**Do you know someone who had/has a liver disease?** (1.4; 1.7)	
Yes	18.8
No	81.2
**Have you ever heard of hepatitis B?** (1.4; 1.7)	
Yes	18.4
No	81.6
**Do you think there is a link between liver disease and hepatitis B?** (1.4; 1.7)	
Yes	8.0
No	92.0
**Do you know what the modes of HBV transmission are?** (0.0; 0.0)	
Sexual transmission	12.2
Contact with blood	14.4
Perinatal transmission	14.3
**Do you know if there is a vaccine that protects against hepatitis B?** (1.4; 1.7)	
Yes	10.7
No	89.3
**HBV knowledge score** (range 0 to 5 points) (0.0; 0.0) ^b^	
Poor knowledge (< 3)	86.7
Good knowledge (≥ 3)	13.3

*Abbreviations*: *HBV* Hepatitis B virus, *HBsAg* Hepatitis B virus surface antigen, *WCBA* Women of childbearing age

^a^Information on household resources including durable goods, agricultural and farming resources was used to build a household index of living conditions using a multiple component analysis

^b^The knowledge score variable was built using the five following items: 1) knowing that there is a link between liver sicknesses and hepatitis B; knowing the three main modes of HBV transmission 2) sexual transmission, 3) contact with blood, 4) perinatal transmission (from mother to child during delivery); 5) knowing that there is vaccine that protects against hepatitis B. One point was awarded for each correct answer and zero for each incorrect or ‘does not know’ answer. The total score ranged from zero to five points

#### Maternal health in WCBA with at least one full-term pregnancy

Most WCBA (97.0%) attended antenatal consultations during their last pregnancy and 71.5% had at least four antenatal consultations (as recommended in Senegal [23]) (Additional file 3). Three quarters (75.5%) of WCBA had given birth at least once in a healthcare facility.

#### Knowledge on hepatitis B

Adult women’s global knowledge of HBV was low (Table 1), with only 18.4% having already heard about hepatitis B, and 10.7% knowing about hepatitis B vaccine. They were also poorly informed about HBV transmission modes, with only 12.2%, 14.4% and 14.3% knowing that the main modes of HBV transmission were sexual intercourse, blood contact and MTCT, respectively. With respect to women aged ≥ 50 years, WCBA tended to be slightly more knowledgeable (*p* = 0.032).

#### History of HBV testing

Only 1.6% of the study population reported having been previously screened for hepatitis B, mostly during pregnancy. All of the latter were aware of their test results (all negative). Among those who had never been screened, 83.3% had never heard of HBV testing and 8.4% had not been offered HBV screening during antenatal consultations. Furthermore, no one who tested HBsAg positive was already aware of their serological status.

### HBsAg positivity prevalence estimations and correlates

#### HBsAg prevalence

**Fig. 2:**
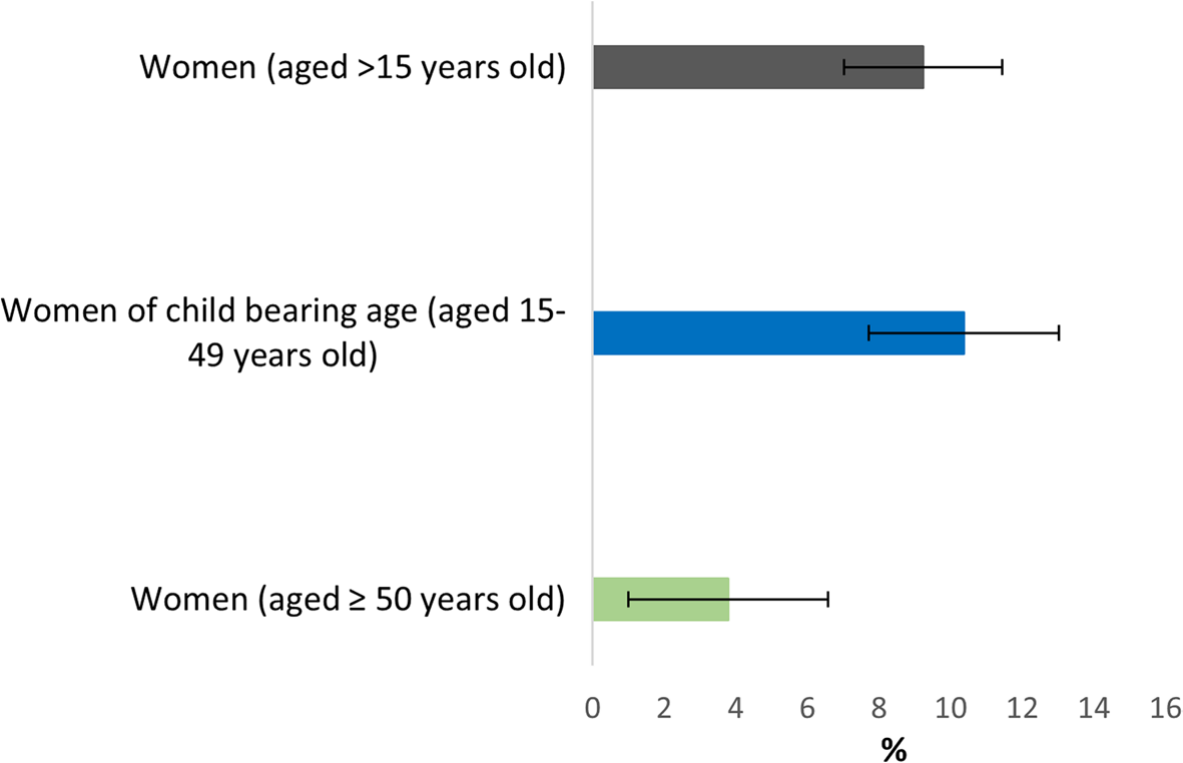
Prevalence of HBsAg positivity in adult women stratified by age in the rural area of Niakhar, Senegal, using weighted and calibrated data (ANRS 12356 AmBASS survey)

Among the 905 adult women, 96 were HBsAg-positive (87 of them were WCBA). HBsAg prevalence in the study population and specifically in WCBA was 9.2% [95% CI 7.0–11.4] and 10.4% [95% CI 7.7–13.0], respectively (Fig. 2, Additional file 4).

#### Profile of HBsAg-positive adult women

Table 2 shows factors associated with HBsAg-positivity in adult women in both univariable and multivariable analyses. In the final multivariable model, at the 10% level, WCBA were five times more at risk of being HBsAg-positive than older women (adjusted odds-ratio (aOR) [95% CI]: 5.15 [1.93–13.78], *p* = 0.004, for 35–49 years and 2.82 [1.09–7.26], *p* = 0.040, for 15–35 years, compared with ≥ 50 years old). This was also the case for adult women who lived in a household with at least two other HBsAg-positive members (aOR [95% CI]: 5.09 [2.88- 9.02], *p* < 0.001), whilst the risk of being HBsAg-positive was twice higher in adult women who knew someone with liver disease (aOR [95% CI]: 2.23 [0.94- 5.27], *p* = 0.065).

**Table 2:** Factors associated with HBsAg-positivity in adult women, logistic regression models using weighted and calibrated data, *n* = 905 (ANRS 12356 AmBASS)

Variables	Univariable analyses (*n* = 905)			Multivariable analysis (*n* = 890)		
	OR	[95% CI]	*P* value	aOR	[95% CI]	*P* value
**Socio-demographic and socioeconomic characteristics**						
**Age group** (years)						
≥ 50 (ref.)	1			1		
> 35 – 49	4.27	[1.75; 10.38]	0.005	5.15	[1.93; 13.78]	0.004
15 – 35	2.52	[1.05; 6.06]	0.041	2.82	[1.09; 7.26]	0.040
**Matrimonial status**						
Married in a polygamous union (ref.)	1					
Married in a monogamous union	0.82	[0.42; 1.60]	0.515			
Not married	0.75	[0.41; 1.35]	0.294			
**Number of children**						
0 (ref.)	1					
1–3	1.66	[0.2; 3.38]	0.140			
≥ 4	1.32	[0.68; 2.55]	0.371			
**Pregnant at the time of the survey**						
No (ref.)	1					
Yes	1.04	[0.33; 3.27]	0.944			
**Area of residence**						
Semi-urban (ref.)	1					
Rural	0.95	[0.52; 1.72]	0.837			
**Educational level**						
Junior high school or above (ref.)	1					
Primary or below	0.73	[0.33; 1.60]	0.385			
Never attended school	1.10	[0.60; 2.01]	0.720			
**Activity**						
Inactive (ref.)	1					
Farming and/or non-farming activity	3.05	[0.67; 13.9]	0.131			
Studies/training	2.00	[0.50; 8.05]	0.289			
**Household wealth index** ^a^						
1^st^ quartile (ref.)	1					
2^nd^ quartile	1.58	[0.61; 4.13]	0.309			
3^rd^ quartile	2.08	[0.91; 4.78]	0.077			
4^th^ quartile	1.40	[0.58; 3.39]	0.417			
**Having community health insurance**						
Yes (ref.)	1					
No	6.36	[0.77;52.57]	0.079			
**Hepatitis b status of household members and knowledge on hepatitis B**						
**Living in a household with at least 2 other HBsAg-positive individuals**						
No (ref.)	1			1		
Yes	4.16	[2.27; 7.62]	< 0.001	5.09	[2.88; 9.02]	< 0.001
**Do you know someone who had/has a liver disease?**						
Yes (ref.)	1			1		
No	2.21	[0.91; 5.38]	0.075	2.23	[0.94; 5.27]	0.065
**Have you ever heard of hepatitis B?**						
Yes (ref.)	1					
No	1.58	[0.78; 3.19]	0.176			
**Do you think there is a link between liver disease and hepatitis B?**						
Yes (ref.)	1					
No	1.65	[0.51; 5.27]	0.357			
**Do you know what the modes of Hepatitis B transmission are?**						
*Sexual transmission*						
Correct response (ref.)	1					
Incorrect	2.41	[0.87; 6.66]	0.082			
*Contact with blood*						
Correct response (ref.)	1					
Incorrect	1.75	[0.77; 3.98]	0.159			
*Perinatal transmission*						
Correct response (ref.)	1					
Incorrect	2.12	[0.85; 5.30]	0.095			
**Do you know if there is a vaccine that protects against hepatitis B?**						
Yes (ref.)	1					
No	1.12	[0.46; 2.76]	0.778			
**Knowledge score** (ranged: 0–5 points) (0.0) ^b^						
Good score (3–5 points)	1					
Poor score (0–2 points)	2.02	[0.79; 5.16]	0.125			

*Abbreviations*: *aOR* Adjusted odds ratio, *CI* confidence interval, *OR* Odds ratio

^a^Information on household resources including durable goods, agricultural and farming resources was used to build a household index of living conditions using a multiple component analysis

^b^The knowledge score variable was built using the five following items: 1) knowing that there is a link between liver sicknesses and hepatitis B; knowing the three main modes of HBV transmission 2) sexual transmission, 3) contact with blood, 4) perinatal transmission (from mother to child during delivery); 5) knowing that there is vaccine that protects against hepatitis B. One point was awarded for each correct answer and zero for each incorrect or ‘does not know’ answer

### HBV MTCT risk and treatment needs

#### HBV MTCT risk in WCBA

Table 3 presents the clinical and biological characteristics of positive-HBsAg WCBA.

**Table 3:** Clinical and biological characteristics of HBsAg-positive women (*n* = 96) using weighted and calibrated data (ANRS 12,356 AmBASS)

**Variables** (% of missing data)	**Adult women (> 15 years)**	**WCBA** **(15–49 years)**
	**(%)or median [IQR]**	
**HBsAg-positive women**	***N* = 96**	***N* = 87**
**HBV DNA (IU/mL)** (0.0; 0.0)		
Undetectable (< 26)	62.5	64.6
26 – 1,999	23.8	21.5
2000 – 19,999	5.5	5.9
20 000 – 199,999	3.2	2.6
> 200 000	5.0	5.4
**HBsAg-positive women who underwent clinical and biological examinations**	***N* = 80**	***N* = 73**
**Ongoing signs of cirrhosis**		
Oedema (0.0; 0.0)	2.1	2.2
Ascites (0.0; 0.0)	1.0	1.1
Icterus (0.0; 0.0)	0.0	0.0
**Family history of hepatocellular carcinoma or cirrhosis in a first degree relative (0.0; 0.0)**	9.3	6.4
**HBeAg-positive** (0.0; 0.0)	8.8	9.4
**HIV positive** (0.0; 0.0)	0.0	0.0
**HDV positive** (0.0;0.0)	0.0	0.0
**ALT**(0.0)^a^		
≤ 19	69.4	68.3
20–38	28.7	29.6
≥ 39	1.9	2.1
**AST** (0.0) ^b^		
< 34	96.1	95.8
34–67	3.9	4.2
≥ 68	0.0	0.0
**Inactive chronic HBV infection** (0.0) HBeAg negative **and** anti-HBe positive **and** HBV DNA < 2,000 IU/mL **and** ALT < ULN^a^	46.9	45.1
**Inactive chronic HBV infection** (0.0) HBeAg negative **and** HBeAg negative **and** anti-HBe positive **and** HBV DNA < 2,000 IU/mL **and** ALT < ULN	46.9	45.1
**APRI** (11.3; 11.0) ^c^		
< 1	97.9	97.7
[1;2]	2.1	2.3
> 2	0.0	0.0
**Eligible for long-term treatment according to the 2015 WHO guidelines**		
APRI > 2 (11.3; 11.0) ^c^	0.0	0.0
Clinical diagnosis of cirrhosis (0.0; 0.0)	0.0	0.0
≥ 30 years old AND abnormal ALT levels^a^ AND HBV DNA > 20,000 IU/ml (0.0; 0.0)	0.0	0.0
**Eligible for antiviral treatment according to national recommendations**		
ALT > 2 × ULN and HBV DNA > 20,000 IU/mL	0.0	0.0
ALT > ULN and HBV DNA > 2,000 IU/mL and FibroScan (at least F2 fibrosis)	0.0	0.0

*Abbreviations*: *HBV DNA* Hepatitis B virus deoxyribonucleic acid, *IU/mL* International units per millilitre, *WCBA* Women of childbearing age, *IQR* Interquartile range, *HBeAg* Hepatitis B virus e antigen, *HDV* Hepatitis D virus, *MTCT* Mother to child transmission, *ALT* Alanine aminotransferase, *AST* Aspartate aminotransferase, *ULN* Upper limit of normal threshold level, *APRI* Aspartate aminotransferase-to-platelet ratio index

^a^ALT: Upper limit of normal threshold level = 19 U/L

^b^AST: Upper limit of normal threshold level = 34 U/L

^c^APRI: AST-to-platelet-ratio-index, APRI = [*(AST/ULN) × 100] / platelet count (10^9^)

HBV DNA was detected (≥ 26 IU/mL) in 35.4% of positive-HBsAg WCBA, 5.9% of whom had HBV DNA levels of 2,000–19,999 IU/mL and 8.0% HBV DNA ≥ 20,000 IU/mL. Additionally, 5.4% of positive-HBsAg WCBA had HBV DNA > 200,000 IU/mL.

#### Need for long-term treatment in adult women in general and specifically in WCBA

The majority of HBsAg-positive adult women (78.6%) agreed to undergo additional clinical and biological examinations. Among them, 8.8% (9.4% of WCBA) were HBeAg-positive.

Approximately 9.3% of HBsAg-positive adult women and 6.4% of WCBA reported a family history of carcinoma or cirrhosis in a first-degree relative, and 3.3% had clinical signs potentially suggestive of the presence of decompensated cirrhosis (oedema, ascites, icterus) but this was not confirmed by laboratory tests (see Table 3).

Less than half (46.9% and 45.1% of all participants and specifically WCBA, respectively) had inactive chronic HBV infection (HBeAg negative and anti-HBe positive and HBV DNA < 2,000 IU/mL and ALT < ULN). None had an APRI score > 2 at the time of the study and none was eligible for long-term treatment.

## Discussion

Our study provides detailed epidemiological data on chronic HBV infection in adult women living in a rural area of Senegal as well as a comprehensive assessment of their needs for HBV prevention and treatment. We found a high prevalence of HBsAg in the study population (9.2% [95% CI: 7.0–11.4]) but a relatively low risk of MTCT in the WCBA subgroup (i.e., 5% in HBsAg-positive WCBA). Half of HBsAg-positive women had active chronic HBV infection but at the time of the study, none were eligible for long-term antiviral treatment according to WHO guidelines [22]. Knowledge of HBV infection was very poor and only 1.6% of the study population had been tested for HBV prior to our study.

### A high HBsAg prevalence in rural Senegalese women

Data on the prevalence of HBsAg in women living in rural areas in West Africa are scarce. Our findings are consistent with two previous studies in Senegal which both estimated the prevalence of HBsAg in pregnant women attending hospitals in Dakar, in 2006–2010 [24] and 2014 [25] at approximately 12%. A meta-analysis in Burkina Faso and a cohort study in Mauritania also reported a similar HBsAg prevalence (i.e., 11.2% and 10.7%, respectively) in pregnant women. However, several studies in The Gambia and Ghana showed a relatively lower HBsAg prevalence than that found by us (specifically, 7.6% and 9.6% in The Gambia [26, 27], and 2.4% to 10.2% in Ghana, depending on the region [ 28–31]). In Nigeria, HBsAg estimations in pregnant women varied from 3.9% to 16.5% over the period 2010–2018 [32–36]. Overall, our findings suggest that rural Senegalese women are one of the subpopulations most affected by chronic HBV infection in West Africa.

### Factors associated with chronic HBV infection

WCBA were more likely to be HBsAg-positive than older women (i.e., ≥ 50 years), which is consistent with several studies conducted in Ghana and Nigeria where young women (aged < 30 years) were at higher risk of chronic HBV infection [28, 33, 34, 36]. “ Decreasing HBsAg prevalence with age could be explained by the presence of occult hepatic infections in older people with chronic HBV infection and a higher all-cause mortality rate in this population [26]. Furthermore, echoing the results of other studies in Nigeria and Ethiopia [37–39], women in our study who were living in a household with at least two other HBsAg-positive members were more likely to be chronically infected with HBV; this suggests the importance of intra-familial transmission. Finally, knowing someone with liver disease was associated with chronic HBV infection. This variable may reflect a greater awareness of HBV infection.

### Low risk of HBV MTCT in rural Senegalese women

Five percent of HBsAg-positive WCBA were at high risk of MTCT. In the only other study which previously measured HBV DNA and HBeAg in pregnant women in Senegal [25], no woman had detectable HBV viral load or positive HBeAg. However, the sample size in that study was small (*n* = 115, with 14 (12%) HBsAg-positive women). Data on HBV MTCT risk are also scarce in SSA, as HBV DNA is rarely assessed. Furthermore, in the three studies that have investigated HBeAg-positivity in pregnant women in Ghana, Ethiopia and Cameroon respectively,[28, 39, 40] estimates for HBeAg-positivity in HBsAg-positive women ranged from 26.1 to 40.0%, which is higher than what we found in our study (approximately 9%).

### No WCBA eligible for long-term treatment

None of the WCBA were eligible for long-term treatment according to WHO’s 2015 recommendations [22], in line with WHO’s statement that “only a small proportion of WCBA would be eligible for long-term treatment” [8]. Furthermore, none of the WCBA were eligible using Senegal’s national recommendations, which are adapted from the European Association for the Study of the Liver (EASL) 2012 clinical practice guidelines [41].

This contrasts with findings from the PROLIFICA study in Senegal, where 10.1% of women > 15 years old were eligible for treatment according to EASL 2012 criteria [42]. However, treatment eligibility according to sex has not been presented by study population (community-based screening, blood bank, hospital-based – only the global estimation is given), and the proportion of women eligible for treatment in the community may therefore be lower.

Of note, more simplified and accurate criteria to identify patients eligible for long-term treatment are needed. Indeed, studies in sub-Saharan Africa have found that WHO simplified criteria do not correctly identify all patients eligible for treatment and have pointed out the complexity of identifying patients when different recommendations exist [43]. For example, in the AmBASS study, treatment eligibility differed in the general population according to the recommendations used (3 men were eligible according to national recommendations and 1 man was eligible according to WHO criteria) [12]. In the PROLIFICA study in Senegal, 9.1% and 1.5% of the community-based study participants were eligible for long-term treatment according to EASL 2012 and WHO recommendations, respectively (18.3% and 5.6% in all study populations) [42]. Furthermore, eligibility to treatment needs to be assessed longitudinally during patients’ follow-up, using non-invasive tests such as APRI or elastometry (Fibroscan®), if available [22].”

### Poor knowledge of HBV infection, low screening rates, and HBV status awareness

Despite the high prevalence of HBsAg positivity, only a small proportion of the study population had basic knowledge about HBV infection and previous experience of HBV screening. Specifically, only 1.6% of women had been previously screened and knew their serological status before our study. The majority reported they had never heard of HBV screening and 8.4% reported they were not offered screening during antenatal consultations although most of them had attended antenatal consultations in the area. Although it is possible that some respondents did not remember HBV infection being talked about or did not remember being offered HBV screening during antenatal consultations, or were screened by health professionals without knowing, these findings suggest that information and communication on HBV infection is insufficient and HBV screening during pregnancy is suboptimal in the study area. A previous study conducted in healthcare workers in the same area highlighted structural barriers to HBV screening, including a lack of healthcare worker training on HBV infection and related counselling, unavailability of RDT, and user fees for HBV screening and treatment [18]. Our findings are in line with previous studies conducted in SSA which also found that pregnant women had poor knowledge of HBV [29, 32, 44], and that levels of screening and HBV status awareness were low [39].

### Health policy recommendations

Our study has important policy implications for the hepatitis B elimination agenda in Senegal as well as for neighboring countries with a similar epidemiologic situation. Knowledge of HBV infection and serological status awareness are key components of HBV prevention and are the first steps of the HBV cascade of care. Given that most women in Senegal attend healthcare facilities during their pregnancy, antenatal consultations represent a major opportunity to give them comprehensive information on HBV infection, its potential consequences on their health and that of their baby, and the importance of being tested [8]. In order for systematic screening to be accessible to all pregnant women in primary healthcare facilities in Senegal, screening should be free and performed using HBsAg RDT, which are highly reliable [45] and deliver results promptly (within 15–45 min); this speed limits the risk of undelivered results and loss-to-follow-up. Furthermore, as chronic HBV infections tend to cluster within families and households in highly endemic areas [46], relatives of HBsAg-positive women should be offered screening for HBV infection.

Finally, despite the low proportion of pregnant women eligible for peripartum antiviral therapy in our study, preventive treatment in high-risk women should be recommended by national programs in high-HBV-prevalence countries, in addition to systematic screening of pregnant women and timely HBV birth dose vaccination in infants [8]. Indeed, HBV birth dose vaccination alone is not sufficient and effective to prevent MTCT as studies in sub-Saharan Africa have highlighted an important residual risk of MTCT despite the timely administration of the HBV birth-dose vaccine [47, 48]. In addition, data from the AmBASS study show that timely coverage of the HBV birth dose vaccine is below WHO recommendations in the Niakhar area (66.8% in 2017–2018) [19].

### Limitations and strengths

Our study has several limitations. First, the Niakhar HDSS area is not representative of the country as a whole. However, it is quite representative of rural areas in Senegal both in terms of socioeconomic and health characteristics of the population and in terms of available health infrastructure and services. Our survey may therefore provide a fair picture of the situation of HBV infection in women in rural Senegal. Second, self-reports of HBV screening history may have been affected by memory bias, which may have led to an underestimation of HBV screening coverage. Third, some information on HBV transmission risk factors (including history of multiple sexual partners, sexually transmitted infections, surgical procedures, abortions and blood transfusion [27, 32–34, 38, 39, 44, 49–53]) were not collected. Therefore, we cannot exclude the risk of bias in the estimation of adjusted odds ratios as we may have omitted potentially relevant covariates in the multivariable model. Fourth, we did not use venous blood sampling, which is the reference method to detect HBsAg but used DBS. However, this method has a high performance with a > 90% diagnostic sensitivity and specificity compared with plasma or serum tests [20]. Furthermore, we conducted a pilot study to determine HBsAg cut-off values (i.e., 1.0 IU/mL for negativity and 1.5 IU/mL for positivity) and all results within this range were systematically confirmed using blood samples. Of the 30 performed on blood samples, 3 were confirmed positive suggesting that the technique used to detect HBsAg is unlikely to change our results.

Despite these weaknesses, our study explored several key issues surrounding HBV control in rural Senegalese women, including chronic HBV infection prevalence and MTCT risk, HBV status awareness, as well as clinical and biological characteristics of women with chronic HBV infection. These data obtained from a large sample of women in rural Senegal can be especially useful for policy makers to design interventions specifically targeted for women in Senegal and in other West African countries facing similar HBV epidemic and health system challenges.

## Conclusions

To conclude, our findings suggest the urgent need for greater HBV prevention and care for women in rural Senegal. They argue for the scale-up of HBV counselling and free screening for women using RDT. Pregnant women should be a priority target. By providing HBV counselling during antenatal consultations, the majority of pregnant women with chronic HBV infection can be identified and provided HBV prevention and care. HBV screening could be extended to their household members to improve the overall HBV cascade of care in rural Senegal [46]. This strategy could greatly help to reach the WHO’s target of HBV elimination by 2030.

## Supplementary Information

**Additional file 1. **Hepatitis B surface antigen (HBsAg) quantified from dried blood spots (DBS) and whole blood samples for participants in the pilot study of the ANRS 12356 AmBASS survey (*n*=30). Table comparing the HBsAg quantified from DBS and whole blood samples.

**Additional file 2. **Methods for data weighting and calibration (ANRS 12356 AmBASS survey). Text describing data weighting and calibration in the AmBASS survey.

**Additional file 3. **Characteristics of women of childbearing age (WCBA) with at least one full-term pregnancy in rural Senegal (ANRS 12356 AmBASS, *n*=439). Table describing the characteristics of WCBA with at least one full-term pregnancy.

**Additional file 4. **Prevalence of HBsAg in adult women stratified by age in the rural area of Niakhar, Senegal (ANRS 12356 AmBASS survey). Table describing the prevalence of HBsAg.

## Data Availability

The datasets analyzed during the current study are not publicly available for the privacy of individuals that participated in the study but are available from the corresponding author on reasonable request.

## Abbreviations

HBV: Hepatitis B virus
HCC: Hepatocellular carcinoma
SSA: Sub-Saharan Africa
MTCT: Mother-to-child transmission
WHO: World Health Organization
HBeAg: Hepatitis B e antigen
CI: Confidence interval
HDSS: Health and Demographic Surveillance System
HBsAg: HBs antigen
WCBA: Women of childbearing age
RDT: Rapid diagnostic tests
ALT: Alanine aminotransferase
AST: Aspartate aminostransferase
HDV: Hepatitis D virus
SD: Standard deviation
APRI: AST-to-platelet ratio index
ULN: Upper limit of normal
DBS: Dried blood spots

## References

[1] Busch K, Thimme R (2015). Natural history of chronic hepatitis B virus infection. Med Microbiol Immunol.

[2] WHO | Global hepatitis report, 2017. WHO. [cited 2020 Aug 19]. Available from: http://www.who.int/hepatitis/publications/global-hepatitis-report2017/en/

[3] Chabrol F, Noah Noah D, Tchoumi EP (2019). Screening, diagnosis and care cascade for viral hepatitis B and C in Yaoundé, Cameroon: a qualitative study of patients and health providers coping with uncertainty and unbearable costs. BMJ Open.

[4] Lemoine M, Thursz MR (2017). Battlefield against hepatitis B infection and HCC in Africa. J Hepatol.

[5] Akinyemiju T, Abera S, Ahmed M, Alam N, Alemayohu MA, Allen C (2017). The Burden of Primary Liver Cancer and Underlying Etiologies From 1990 to 2015 at the Global, Regional, and National Level. JAMA Oncol.

[6] Shimakawa Y, Lemoine M, Njai HF (2016). Natural history of chronic HBV infection in West Africa: A longitudinal population-based study from The Gambia. Gut.

[7] WHO. Global health sector strategy on viral hepatitis 2016–2021. Geneva: WHO, 2016. Available at: https://www.who.int/hepatitis/strategy2016-2021/ghss-hep/en/. Accessed 10 Aug 2020.

[8] Prevention of mother-to-child transmission of hepatitis B virus: Guidelines on antiviral prophylaxis in pregnancy. [cited 2020 Aug 19]. Available from: https://www.who.int/publications-detail-redirect/978-92-4-000270-8 32833415

[9] Polaris Observatory Collaborators (2018). Global prevalence, treatment, and prevention of hepatitis B virus infection in 2016: a modelling study. Lancet Gastroenterol Hepatol.

[10] Edmunds WJ, Medley GF, Nokes DJ (1996). Epidemiological patterns of hepatitis B virus (HBV) in highly endemic areas. Epidemiol Infect.

[11] Nayagam S, Shimakawa Y, Lemoine M (2020). Mother-to-child transmission of hepatitis B: What more needs to be done to eliminate it around the world?. J Viral Hepat.

[12] Périères L, Diallo A, Marcellin F, Nishimwe ML, Ba EH, Coste M (2022). Hepatitis B in Senegal: A Successful Infant Vaccination Program but Urgent Need to Scale Up Screening and Treatment (ANRS 12356 AmBASS survey). Hepatol Commun.

[13] Programme National de Lutte Contre les Hépatites (2019). Strategic plan against viral hepatitis in Senegal (2019–2023): Policy brief.

[14] Bertoletti A, Kennedy PT (2015). The immune tolerant phase of chronic HBV infection: new perspectives on an old concept”. Cell Mol Immunol..

[15] Lavanchy D (2004). Hepatitis B virus epidemiology, disease burden, treatment, and current and emerging prevention and controlmeasures. J Viral Hepatitis.

[16] Coste M, De Sèze M, Diallo A, Carrieri MP, Marcellin F, Boyer S. Burden and impacts of chronic hepatitis B infection in rural Senegal: study protocol of a cross-sectional survey in the area of Niakhar (AmBASS ANRS 12356). BMJ Open. [cited 2020 Jul 7]. 2019;9(7). Available from: https://www.ncbi.nlm.nih.gov/pmc/articles/PMC6661601/10.1136/bmjopen-2019-030211 PMC6661601 31320358

[17] Delaunay V, Douillot L, Diallo A (2013). Profile: The Niakhar Health and Demographic Surveillance System. Int J Epidemiol.

[18] Djaogol T, Coste M, Marcellin F, Jaquet A, Chabrol F, Giles-Vernick T, Diallo A, Carrieri MP, Boyer S, ANRS 12356 AmBASS Study Group (2019). Prevention and care of hepatitis B in the rural region of Fatick in Senegal: a healthcare workers’ perspective using a mixed methods approach. BMC Health Serv Res..

[19] Périères L, Marcellin F, Lo G, Protopopescu C, Ba EH, Coste M (2021). Hepatitis B Vaccination in Senegalese Children: Coverage, Timeliness, and Sociodemographic Determinants of Non-Adherence to Immunisation Schedules (ANRS 12356 AmBASS Survey). Vaccines.

[20] WHO | Guidelines on hepatitis B and C testing, February 2017. WHO. [cited 21 Feb 2018]. Available on: http://www.who.int/hepatitis/publications/guidelines-hepatitis-c-b-testing/en/.

[21] Mohamed S, Raimondo A, Pe´naranda G, Camus C, Ouzan D,  (2013). Dried Blood Spot Sampling for Hepatitis B Virus Serology and Molecular Testing. PLoS ONE.

[22] WHO | Guidelines for the prevention, care and treatment of persons with chronic hepatitis B infection, March 2015. WHO. [Cited 21 Feb 2018]. Available on: http://www.who.int/hepatitis/publications/hepatitis-b-guidelines/en/. 26225396

[23] Agence Nationale de la Statistique et de la Démographie - Situation Economique et Sociale du Sénégal -dakar2013.pdf. [cited 2020 Aug 20]. Available from: http://www.ansd.sn/ressources/ses/chapitres/3-sante-dakar2013.pdf

[24] Lo G, Diawara PS, Diouf NN, Faye B, Seck MC, Sow K (2012). Prévalence de l’antigène de surface du virus de l’hépatite B (AgHBs) chez les femmes enceintes au laboratoire de l’Hôpital Militaire de Ouakam (HMO). Dakar Med Afr Noire.

[25] Niang MS, Fall KS, Mbengue B, Mbow M, Diouf NN, Boye O (2017). Immunological Status to Hepatitis B Virus of Pregnant Women in Dakar. Senegal Open J Immunol.

[26] Lemoine M, Shimakawa Y, Njie R, Taal M, Ndow G, Chemin I (2016). Acceptability and feasibility of a screen-and-treat programme for hepatitis B virus infection in The Gambia: the Prevention of Liver Fibrosis and Cancer in Africa (PROLIFICA) study. Lancet Glob Health.

[27] Bittaye M, Idoko P, Ekele BA, Obed SA, Nyan O (2019). Hepatitis B virus sero-prevalence amongst pregnant women in the Gambia. BMC Infect Dis.

[28] Luuse A, Dassah S, Lokpo S, Ameke L, Noagbe M, Adatara P (2016). Sero-Prevalence of Hepatitis B Surface Antigen Amongst Pregnant Women Attending an Antenatal Clinic, Volta Region, Ghana. J Public Health Afr.

[29] Adjei CA, Atibila F, Apiribu F, Ahordzor F, Attafuah PA, Ansah-Nyarko M (2018). Hepatitis B Infection among Parturient Women in Peri-Urban Ghana. Am J Trop Med Hyg.

[30] Dortey BA, Anaba EA, Lassey AT, Damale NKR, Maya ET (2020). Seroprevalence of Hepatitis B virus infection and associated factors among pregnant women at Korle-Bu Teaching Hospital, Ghana. PLoS ONE.

[31] Ephraim R, Donko I, Sakyi SA, Ampong J, Agbodjakey H (2015). Seroprevalence and risk factors of Hepatitis B and Hepatitis C infections among pregnant women in the Asante Akim North Municipality of the Ashanti region, Ghana; a cross sectional study. Afr Health Sci.

[32] Atilola G, Tomisin O, Randle M, Isaac KO, Odutolu G, Olomu J (2018). Epidemiology of HBV in Pregnant Women, South West Nigeria. J Epidemiol Glob Health.

[33] Utoo BT (2013). Hepatitis B surface antigenemia (HBsAg) among pregnant women in southern Nigeria. Afr Health Sci.

[34] Anaedobe CG, Fowotade A, Omoruyi CE, Bakare RA (2015). Prevalence, sociodemographic features and risk factors of Hepatitis B virus infection among pregnant women in Southwestern Nigeria. Pan Afr Med J.

[35] Omatola CA, Lawal C, Omosayin DO, Okolo MLO, Adaji DM, Mofolorunsho CK (2019). Seroprevalence of HBV, HCV, and HIV and Associated Risk Factors Among Apparently Healthy Pregnant Women in Anyigba. Nigeria Viral Immunol.

[36] Aba HO, Aminu M (2016). Seroprevalence of hepatitis B virus serological markers among pregnant Nigerian women. Ann Afr Med.

[37] Adegbesan-Omilabu MA, Okunade KS, Gbadegesin A, Olowoselu OF, Oluwole AA, Omilabu SA (2015). Seroprevalence of hepatitis B virus infection among pregnant women at the antenatal booking clinic of a Tertiary Hospital in Lagos Nigeria. Niger J Clin Pract.

[38] Araya Mezgebo T, Niguse S, Gebrekidan Kahsay A, Hailekiros H, Berhe N, Asmelash DT (2018). Hepatitis B virus infection and associated risk factors among pregnant women attending antenatal care in health facilities of Tigray. Northern Ethiopia J Med Virol.

[39] Amsalu A, Ferede G, Eshetie S, Tadewos A, Assegu D (2018). Prevalence, Infectivity, and Associated Risk Factors of Hepatitis B Virus among Pregnant Women in Yirgalem Hospital, Ethiopia: Implication of Screening to Control Mother-to-Child Transmission. J Pregnancy.

[40] Sone LHE, Voufo RA, Dimodi HT, Kengne M, Gueguim C, Ngah N (2017). Prevalence and Identification of Serum Markers Associated with Vertical Transmission of Hepatitis B in Pregnant Women in Yaounde. Cameroon Int J MCH AIDS.

[41] European Association for the Study of the Liver (2012). EASL Clinical Practice Guidelines: Management of chronic hepatitis B virus infection. J Hepatol.

[42] Sow A, Lemoine M, Toure PS, Diop M, Lo G, De Veiga J (2022). HBV continuum of care using community- and hospital-based screening interventions in Senegal: Results from the PROLIFICA programme. JHEP Rep Innov Hepatol.

[43] Aberra H, Desalegn H, Berhe N, Mekasha B, Medhin G, Gundersen SG (2019). The WHO guidelines for chronic hepatitis B fail to detect half of the patients in need of treatment in Ethiopia. J Hepatol.

[44] Ngaira JAM, Kimotho J, Mirigi I, Osman S, Ng’ang’a Z, Lwembe R (2016). Prevalence, awareness and risk factors associated with Hepatitis B infection among pregnant women attending the antenatal clinic at Mbagathi District Hospital in Nairobi. Kenya. Pan Afr Med J..

[45] Amini A, Varsaneux O, Kelly H, Tang W, Chen W, Boeras DI, Falconer J, Tucker JD, Chou R, Ishizaki A, Easterbrook P, Peeling RW (2017). Diagnostic accuracy of tests to detect hepatitis B surface antigen: a systematic review of the literature and meta-analysis. BMC Infect Dis.

[46] Dumpis U, Holmes EC, Mendy M, Hill A, Thursz M, Hall A, Whittle H, Karayiannis P (2001). Transmission of hepatitis B virus infection in Gambian families revealed by phylogenetic analysis. J Hepatol.

[47] Shimakawa Y, Veillon P, Birguel J, Pivert A, Sauvage V, Guillou-Guillemette HL (2022). Residual risk of mother-to-child transmission of hepatitis B virus infection despite timely birth-dose vaccination in Cameroon (ANRS 12303): a single-centre, longitudinal observational study. Lancet Glob Health.

[48] Ekra D, Herbinger KH, Konate S, Leblond A, Fretz C, Cilote V (2008). A non-randomized vaccine effectiveness trial of accelerated infant hepatitis B immunization schedules with a first dose at birth or age 6 weeks in Côte d’Ivoire. Vaccine.

[49] Chernet A, Yesuf A, Alagaw A (2017). Seroprevalence of Hepatitis B virus surface antigen and factors associated among pregnant women in Dawuro zone, SNNPR, Southwest Ethiopia: a cross sectional study. BMC Res Notes.

[50] Mansour W, Malick FZF, Sidiya A, Ishagh E, Chekaraou MA, Veillon P (2012). Prevalence, risk factors, and molecular epidemiology of hepatitis B and hepatitis delta virus in pregnant women and in patients in Mauritania. J Med Virol.

[51] Manyahi J, Msigwa Y, Mhimbira F, Majigo M (2017). High sero-prevalence of hepatitis B virus and human immunodeficiency virus infections among pregnant women attending antenatal clinic at Temeke municipal health facilities, Dar es Salaam, Tanzania: a cross sectional study. BMC Pregnancy Childbirth..

[52] Kirbak ALS, Ng’ang’a Z, Omolo J, Idris H, Usman A, Mbabazi WB (2017). Sero-prevalence for Hepatitis B virus among pregnant women attending antenatal clinic in Juba Teaching Hospital, Republic of South Sudan. Pan Afr Med J..

[53] Kolawole OM, Wahab AA, Adekanle DA, Sibanda T, Okoh AI (2012). Seroprevalence of hepatitis B surface antigenemia and its effects on hematological parameters in pregnant women in Osogbo, Nigeria. Virol J.

