# Supplementary material for: Hepatitis B prevention and treatment needs in women in Senegal (ANRS 12356 AmBASS survey)
Source: BMC Public Health. 2023 May 5;23:825. doi: 10.1186/s12889-023-15710-y (PMC10161542; doi:10.1186/s12889-023-15710-y)
Supplement: Supplementary file 1 — Additional file 1. Hepatitis B surface antigen (HBsAg) quantified from dried blood spots (DBS) and whole blood samples for participants in the pilot study of the ANRS 12356 AmBASS survey (n=30). Table comparing the HBsAg quantified from DBS and whole blood samples. [file 12889_2023_15710_MOESM1_ESM.docx]

**Additional file 1.** **Hepatitis B surface antigen (HBsAg) quantified from dried blood spots (DBS) and whole blood samples for participants in the pilot study of the ANRS 12356 AmBASS survey** (n=30)

| *HBsAg on DBS (OD)* † | *HBsAg on Serum (S/CO)* |
| --- | --- |
| 0.93 | 0.40 |
| 0.83 | 0.41 |
| 0.96 | 0.41 |
| 0.81 | 0.42 |
| 0.84 | 0.47 |
| 0.73 | 0.56 |
| 0.96 | 0.48 |
| 0.87 | 0.42 |
| 0.72 | 0.41 |
| 0.88 | 0.49 |
| 0.96 | 0.45 |
| 0.84 | 0.46 |
| 0.88 | 0.45 |
| 0.68 | 0.59 |
| 0.82 | 0.39 |
| 0.99 | 0.79 |
| 0.91 | 0.45 |
| 0.79 | 0.55 |
| 0.74 | 0.50 |
| 0.68 | 0.55 |
| 0.91 | 0.43 |
| 0.98 | 0.45 |
| 0.89 | 0.97 |
| 0.90 | 0.56 |
| 0.82 | 0.49 |
| 1.17 | 0.70 |
| 1.43 | 0.86 |
| 2918.37 | 1237.48 |
| 1259.78 | 2933.78 |
| 1263.61 | 9735.50 |

| † DBS obtained using capillary whole blood. |
| --- |

- Positive serum when S/CO>1
- OD: optical density
