# Supplementary material for: Hepatitis B prevention and treatment needs in women in Senegal (ANRS 12356 AmBASS survey)
Source: BMC Public Health. 2023 May 5;23:825. doi: 10.1186/s12889-023-15710-y (PMC10161542; doi:10.1186/s12889-023-15710-y)
Supplement: Supplementary file 2 — Additional file 2. Methods for data weighting and calibration (ANRS 12356 AmBASS survey). Text describing data weighting and calibration in the AmBASS survey. [file 12889_2023_15710_MOESM2_ESM.docx]

**Additional file 2.** **Methods for data weighting and calibration (ANRS 12356 AmBASS survey)**

Sampling weights were calculated as the inverse of the individual probability of inclusion in the sample, divided by the number of months each individual in the household was present during the preceding year. The following variables were used: number of participants in the household, number of eligible individuals in the household, number of households participating in the survey in the given village, number of randomly selected households in the given village, number of villages randomly selected for the survey, number of villages in the Niakhar HDSS.

We hypothesised that children were continuously present in their household during the previous 12 months. Final weights were obtained after multiplying sampling weights by calibration factors, calculated as the ratio of the percentage of individuals in the HDSS demographic database to the percentage of individuals in the survey sample, for each age-sex class.
