# Supplementary material for: Hepatitis B prevention and treatment needs in women in Senegal (ANRS 12356 AmBASS survey)
Source: BMC Public Health. 2023 May 5;23:825. doi: 10.1186/s12889-023-15710-y (PMC10161542; doi:10.1186/s12889-023-15710-y)
Supplement: Supplementary file 3 — Additional file 3. Characteristics of women of childbearing age (WCBA) with at least one full-term pregnancy in rural Senegal (ANRS 12356 AmBASS, n=439). Table describing the characteristics of WCBA with at least one full-term pregnancy. [file 12889_2023_15710_MOESM3_ESM.docx]

**Additional file 3.** **Characteristics of women of childbearing age (WCBA) with at least one full-term pregnancy in rural Senegal (ANRS 12356 AmBASS, n=439)**

| **VARIABLES** (% of missing) | | **No. of women**  **(%) or mean (SD)** |
| --- | --- | --- |
| **SOCIO-DEMOGRAPHIC AND SOCIOECONOMIC CHARACTERISTICS** | | |
| **Age** (in years) (0.0) | | 34.1 (8.6) |
| **Matrimonial status** (0.3) | |  |
|  | Married in a polygamous union | 72.3 |
|  | Married in a monogamous union | 22.5 |
|  | Not married | 5.2 |
| **Total number of children**  (0.3) | | 5.0 (3.0) |
| **Number of children** (0.3) | |  |
|  | 0 | 0.3 |
|  | 1-3 | 37.5 |
|  | ≥4 | 62.2 |
| **Area of residence** (0.0) | |  |
|  | Semi-urban | 37.7 |
|  | Rural | 62.3 |
| **Educational level** (1.1) | |  |
|  | High school or above | 10.8 |
|  | Primary or junior high school | 19.0 |
|  | Never attended school | 70.2 |
| **Activity** (0.0) | |  |
|  | Farming and/or non-farming activity | 7.1 |
|  | Studies/training | 86.5 |
|  | Inactive | 6.4 |
| **Household index of life conditions** (0.0) † | |  |
|  | 1^st^ quartile | 16.5 |
|  | 2^nd^ quartile | 21.8 |
|  | 3^rd^ quartile | 22.6 |
|  | 4^th^ quartile | 39.1 |
| **Having community health insurance** (0.0) | |  |
|  | Yes | 5.9 |
|  | No | 94.1 |
| **HBV STATUS AT THE TIME OF THE AMBASS SURVEY AND HBV SCREENING HISTORY** | | |
| **Positive HBsAg** (0.0) | | 12.5 |
| **Living in a household with more than two HBsAg-positive individuals** (0.0) | | 5.9 |
| **Having been previously tested for HBV** (0.2) | |  |
|  | Yes | 3.2 |
|  | No | 96.8 |
| **Awareness of HBV serological status among women previously tested** (0.0) | | N=7 |
|  | Yes, positive | 100.0 |
|  | Yes, negative | 0.0 |
|  | No (did not know) | 0.0 |
| **Reasons for never having been tested for HBV** | | N=426 |
|  | Never heard of HBV testing (3.3) | 74.4 |
|  | Not offered in antenatal sessions (3.3) | 16.3 |
| **GLOBAL KNOWLEDGE ON HEPATITIS B** | | |
| **Are you aware of the liver diseases, which we call ‘big belly’ and ‘yellow eyes’?** (0.0) | |  |
|  | Yes | 23.7 |
|  | No | 76.3 |
| **Do you know someone who had/has a liver disease?** (0.0) | |  |
|  | Yes | 20.9 |
|  | No | 79.1 |
| **Have you ever heard of hepatitis B?** (0.0) | |  |
|  | Yes | 14.2 |
|  | No | 85.8 |
| **Do you think there is a link between liver sicknesses and hepatitis B?** (0.0) | |  |
|  | Yes | 6.1 |
|  | No | 93.9 |
| **Do you know what the modes of HBV transmission are?** (0.0) | |  |
|  | Sexual transmission | 9.7 |
|  | Contact with blood | 11.1 |
|  | Perinatal transmission | 11.5 |
| **Do you know if there is a vaccine that protects against hepatitis B** (0.0) | |  |
|  | Yes | 10.2 |
|  | No | 89.8 |
| **HBV knowledge score** (range 0 to 5 points) (0.0) ‡ | |  |
|  | Poor knowledge (<3) | 89.3 |
|  | Good knowledge (>=3) | 10.7 |
| **MATERNAL HEALTH CHARACTERISTICS (DATA FOR THE LAST FULL-TERM PREGNANCY)** | | |
| **Total number of full-term pregnancies** (4.2) | | 5.0 (3.0) |
| **Pregnant at the time of the survey** (0.0) | |  |
|  | No | 91.1 |
|  | Yes | 8.9 |
| **Delivery by caesarian section** (3.7) | |  |
|  | Yes | 5.4 |
|  | No | 94.6 |
| **Place of delivery** (4.3) | |  |
|  | In healthcare facility | 75.3 |
|  | At home | 24.7 |
| **Number of antenatal consultations received** (8.6) | |  |
|  | <4 | 25.5 |
|  | ≥4 | 71.5 |
| **People who provided assistance during delivery** (2.4) | |  |
|  | Family / friend | 22.2 |
|  | Traditional midwife | 1.2 |
|  | Matron | 18.4 |
|  | Assistant midwife / assistant nurse | 20.8 |
|  | Midwife / State nurse | 35.6 |
|  | Doctor | 1.8 |
| **CLINICAL AND BIOLOGICAL CHARACTERISTICS OF HBSAG-POSITIVE WOMEN** | | |
| **HBsAg-positive women** | | ***n=64*** |
| **HBV DNA (IU/mL)** (0.0) | |  |
|  | Undetectable (HBV DNA <26 IU/mL) | 69.4 |
|  | Viral load 26 IU/mL - 2000 IU/mL | 16.4 |
|  | Viral load >2000 – 20 000 IU/mL | 6.9 |
|  | Viral load >20 000 – 200 000 IU/mL | 3.7 |
|  | Viral load >200 000 IU/mL | 3.5 |
| **HBsAg-positive women who underwent clinical and biological examination** | | **N=54** |
| **Ongoing signs of cirrhosis** | |  |
|  | Oedema (0.0)  Ascites (0.0)  Icterus (0.0) | 3.1  1.5  0.0 |
| **Family history of hepatocellular carcinoma or cirrhosis in a first degree relative (0.0)** | | 9.2 |
| **HBeAg-positive (0.0)** | | 0.0 |
| **ALT(0.0)**  <40  40-79  ≥80 | | 95.7  2.8  1.5 |
| **AST (0.0)**  <34  34-67  ≥ 68 | | 97.1  2.9  0.0 |
| **Inactive chronic HBV infection (0.0)**  **HBeAg negative and anti-HBe positive and HBV DNA <2,000 IU/mL and ALT<ULN** | | 43.5 |
| **Active chronic HBV infection (0.0)**  **HBeAg positive or anti-HBe negative or HBV DNA ≥2,000 IU/mL or ALT>ULN** | | 56.5 |
| **APRI** (9.3) | <1  [1 ;2]  >2 | 95.9  4.1  0.0 |
| **Eligible for long-term treatment according to the 2015 WHO guidelines** | | 0.0 |
|  | APRI >2 (9.3) | 0.0 |
|  | Clinical diagnosis of cirrhosis (0.0) | 0.0 |
|  | ≥30 years old AND abnormal ALT levels^‡^ AND HBV DNA>20,000 IU/ml (0.0) | 0.0 |

HBV= hepatitis B virus; HBsAg= Hepatitis B virus surface antigen; WCBA=women of childbearing age.

† Information on household resources including durable goods, agricultural and farming resources was used to build a household index of living conditions using a multiple component analysis.

‡ The knowledge score variable was built using the five following items: 1) knowing that there is a link between liver sicknesses and hepatitis B; knowing the three main modes of HBV transmission 2) sexual transmission, 3) contact with blood, 4) perinatal transmission (from mother to child during delivery); 5) knowing that there is vaccine that protects against hepatitis B. One point was awarded for each correct answer, and zero for each incorrect or ‘does not know’ answer. The total score ranged from zero to five points.
