# Supplementary material for: Hepatitis B prevention and treatment needs in women in Senegal (ANRS 12356 AmBASS survey)
Source: BMC Public Health. 2023 May 5;23:825. doi: 10.1186/s12889-023-15710-y (PMC10161542; doi:10.1186/s12889-023-15710-y)
Supplement: Supplementary file 4 — Additional file 4. Prevalence of HBsAg in adult women stratified by age in the rural area of Niakhar, Senegal (ANRS 12356 AmBASS survey). Table describing the prevalence of HBsAg. [file 12889_2023_15710_MOESM4_ESM.docx]

**Additional file 4.** **Prevalence of HBsAg in adult women stratified by age in the rural area of Niakhar, Senegal (ANRS 12356 AmBASS survey)**

|  | **No. of chronic HBV carriers/ total no. of individuals** | **Prevalence of chronic hepatitis B**  **[95% CI]** | |
| --- | --- | --- | --- |
|  |  | **Weighted and calibrated data** | **Crude data** |
| **Adult**  **women (** aged >15 years old) | 96/905 | 9.2 [7.0; 11.4] | 10.6 [8.6; 12.6] |
| **Women of childbearing age** (aged 15-49 years old) | 87/720 | 10.4 [7.7; 13.0] | 12.1 [9.7; 14.5] |
| **Older women (** aged >=50 years old) | 9/185 | 3.8 [1.0; 6.6] | 4.9 [1.8; 8.0] |

Abbreviations: CI = Confidence Intervals; HBV = Hepatitis B virus.
